# Losses of Both Products of the *Cdkn2a/Arf* Locus Contribute to Asbestos-Induced Mesothelioma Development and Cooperate to Accelerate Tumorigenesis

**DOI:** 10.1371/journal.pone.0018828

**Published:** 2011-04-19

**Authors:** Deborah A. Altomare, Craig W. Menges, Jinfei Xu, Jianming Pei, Lili Zhang, Ara Tadevosyan, Erin Neumann-Domer, Zemin Liu, Michele Carbone, Ilse Chudoba, Andres J. Klein-Szanto, Joseph R. Testa

**Affiliations:** 1 Cancer Biology Program, Fox Chase Cancer Center, Philadelphia, Pennsylvania, United States of America; 2 Thoracic Oncology Program, Cancer Research Center of Hawaii, University of Hawaii, Honolulu, Hawaii, United States of America; 3 MetaSystems, Altlussheim, Germany; University of Texas M. D. Anderson Cancer Center, United States of America

## Abstract

The *CDKN2A/ARF* locus encompasses overlapping tumor suppressor genes *p16(INK4A)* and *p14(ARF)*, which are frequently co-deleted in human malignant mesothelioma (MM). The importance of *p16(INK4A)* loss in human cancer is well established, but the relative significance of *p14(ARF)* loss has been debated. The tumor predisposition of mice singly deficient for either *Ink4a* or *Arf*, due to targeting of exons 1α or 1β, respectively, supports the idea that both play significant and nonredundant roles in suppressing spontaneous tumors. To further test this notion, we exposed *Ink4a*(+/−) and *Arf*(+/−) mice to asbestos, the major cause of MM. Asbestos-treated *Ink4a*(+/−) and *Arf*(+/−) mice showed increased incidence and shorter latency of MM relative to wild-type littermates. MMs from *Ink4a*(+/−) mice exhibited biallelic inactivation of *Ink4a*, loss of Arf or p53 expression and frequent loss of p15(Ink4b). In contrast, MMs from *Arf*(+/−) mice exhibited loss of Arf expression, but did not require loss of Ink4a or Ink4b. Mice doubly deficient for *Ink4a* and *Arf*, due to deletion of *Cdkn2a/Arf* exon 2, showed accelerated asbestos-induced MM formation relative to mice deficient for *Ink4a* or *Arf* alone, and MMs exhibited biallelic loss of both tumor suppressor genes. The tumor suppressor function of Arf in MM was p53-independent, since MMs with loss of Arf retained functional p53. Collectively, these *in vivo* data indicate that both *CDKN2A/ARF* gene products suppress asbestos carcinogenicity. Furthermore, while inactivation of Arf appears to be crucial for MM pathogenesis, the inactivation of both *p16(Ink4a)* and *p19(Arf)* cooperate to accelerate asbestos-induced tumorigenesis.

## Introduction

*CDKN2A/ARF* is among the most commonly mutated loci in human cancer, encoding two different tumor suppressors translated from alternatively spliced mRNAs. *p16(INK4A)* is composed of exons 1α, 2 and 3, and is designated here as *INK4A* (inhibitor of cyclin dependent kinase 4). Human *p14(ARF)* is encoded by exon 1β and alternate reading frames of *CDKN2A/ARF* exons 2 and 3, herein referred to as *ARF* (alternate reading frame). Knockout mice with targeted deletion of specific *Cdkn2A/Arf* exons have disrupted *p16Ink4a*, *p19Arf* or both genes [1], [2], [3], [4] and develop a different spectrum of spontaneous tumors, although not malignant mesotheliomas (MMs). A differential impact of heterozygous loss of *Ink4a* or *Arf* to the induction of MM by asbestos has not been previously addressed. The studies presented here provide genetic evidence for the significance of *Ink4a* and *Arf* alterations in MM by directly comparing susceptibility to tumor induction by asbestos in *Ink4a*-deficient, *Arf*-deficient and doubly heterozygous *Ink4a*;*Arf* mice in a common genetic background. The enhanced tumor susceptibility of mice singly deficient for either *p16(Ink4a)* or *p19(Arf)* supports the view that both play significant and nonredundant roles in suppressing malignant transformation. The fact that mice deficient for both tumor suppressors have accelerated tumor development indicates that inactivation of both *p16(Ink4a)* and *p19(Arf)* cooperate to promote asbestos carcinogenicity.

## Results

**Figure 1:**
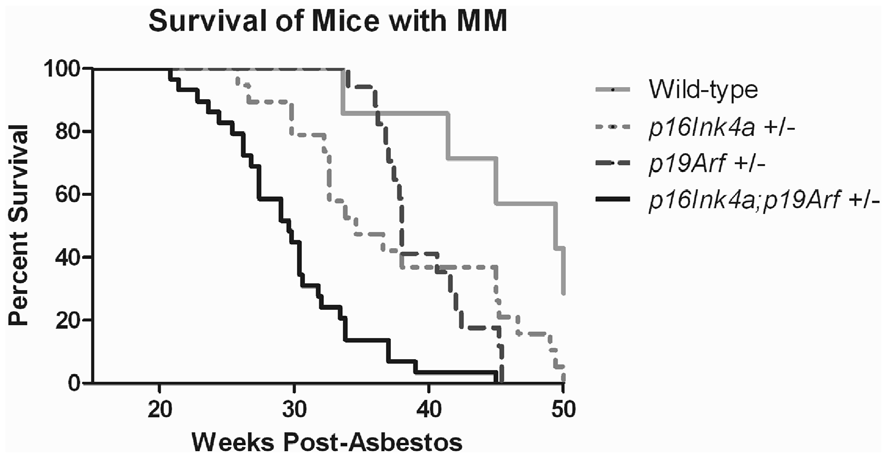
Accelerated asbestos-induced MM in mice deficient for *Ink4a*, *Arf*, or *Ink4a*;*Arf*. Kaplan-Meier survival curves (GraphPad Prism 5). Mice with no tumors or with incidental tumors were censored. A log-rank test verified significant differences between wild-type and *Ink4a*-, *Arf*- and *Ink4a*;*Arf*-deficient groups (p-value = 0.0342, 0.0312, and <0.0001, respectively).

Using a genetic approach, we assessed the relative contribution of Ink4a and Arf deficiency to induction of asbestos-induced tumor formation. MM incidence was increased and latency decreased in *Ink4a*(+/−) and *Arf*(+/−) mice relative to wild-type littermates (Fig. 1, Supporting File S1). Mice doubly deficient for *Ink4a*;*Arf* had accelerated asbestos-induced MMs relative to mice deficient for *Ink4a* or *Arf* alone. As in prior studies [5], [6], [7], MM did not develop in TiO_2_-treated mice (Supporting File S1). Median latency for MM detection after initial asbestos exposure was 29.6 weeks for *Ink4a*;*Arf*(+/−), 34.6 weeks for *Ink4a*(+/−), 38.0 weeks for *Arf*(+/−), and 49.4 weeks for wild-type mice. *Ink4a*-, *Arf- and Ink4a*;*Arf*-deficient mice had decreased survival compared to wild-type mice (Fig. 1). Tumor latency for *Ink4a*- and *Arf*-deficient mice was not significantly different, whereas latency was decreased in *Ink4a*;*Arf*-deficient mice (p<0.0001).

**Figure 2:**
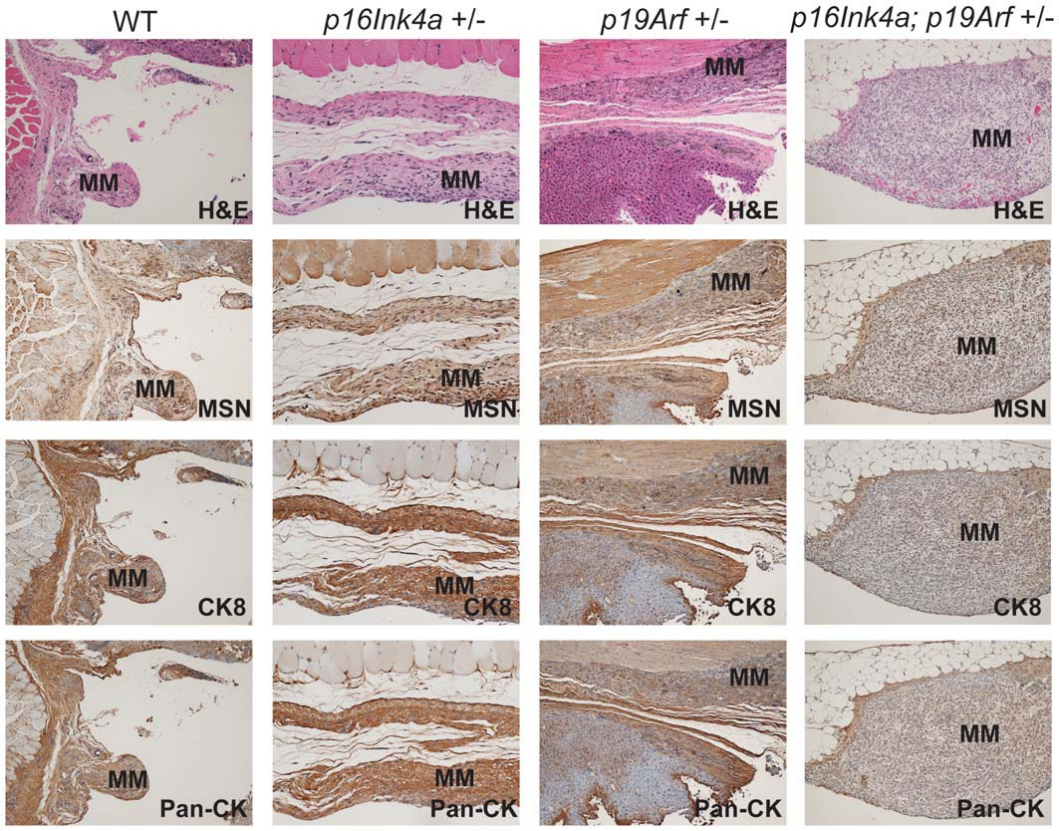
Representative histopathology of asbestos-induced MMs. Sections were stained with hematoxylin and eosin (H&E) or with antibodies against mesothelin (MSN), cytokeratin (CK8), and pan-cytokeratin (Pan-CK).

Sarcomatoid MMs were prevalent in all asbestos-treated mice (Fig. 2), although biphasic and epithelial morphology were occasionally observed. MMs frequently presented with ascites, occasional spheroids, and diffuse peritoneal seeding of the serosal lining. Most tumors in wild-type mice were early-stage MMs, whereas tumors in *Ink4a*(+/−), *Arf*(+/−), and *Ink4a*;*Arf*(+/−) mice generally were more advanced. Extensive disease was most evident in doubly heterozygous mice. In a few cases, diagnosis was difficult because MMs may arise anywhere in the abdominal cavity and may not be obvious in the sampled tissues. Thus, where possible, tumor cells from ascites or peritoneal lavage were tested with markers to verify MM (Supporting File S1).

**Figure 3:**
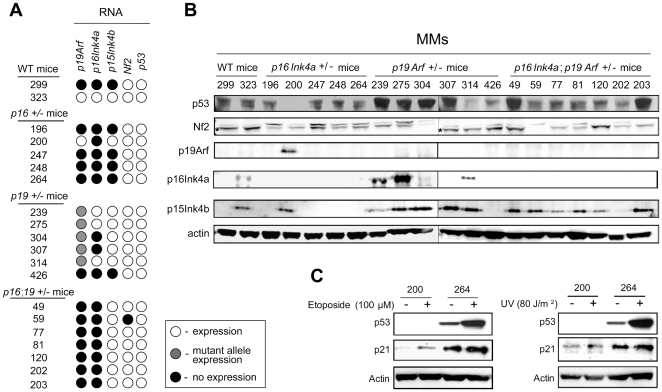
Biallelic inactivation of predisposing *Ink4a* and *Arf* tumor suppressor genes in MM. *A*, Composite depicting RT-PCR for *Arf* exon 1β, *Ink4a* exon 1α, *Ink4b*, *Nf2* and *Tp53* from primary MM cells (passage ≤5). *B*, Immunoblotting of MM cells. Note that Arf was lost in nearly all tumors, with p53 expression being absent in the only tumor (#200) retaining Arf expression. Two bands were observed in the Nf2 immunoblot (panel 3). An asterisk beside the lower band depicts Nf2, with a non-specific band accounting for the upper signal. Actin was a loading control. *C*, Immunoblot of MM cells treated with Etoposide (100 µM) or UV irradiation (80 J/m^2^) 24 hours post-treatment.

Using early passage (≤6) MM cells, we found biallelic infactivation of the predisposing tumor suppressor gene in all MMs tested (Fig. 3A). In most (4 of 5 tested) MMs from *Ink4a*(+/−) mice, there also was loss of *Arf* and *p15(Ink4b)*, the latter located near the *Cdkn2a/Arf* locus. Tumor #200, from an *Ink4a*(+/−) mouse, retained expression of Arf but did not express p53 protein. Interestingly, in MM cells with loss of Arf, the p53 pathway appeared to remain functional intact based on their response to DNA damage with etoposide (100 µM) or UV irradiation (80 J/m^2^) (Fig. 3C).

MMs from *Arf*(+/−) mice consistently exhibited loss of Arf protein. In 4 of 5 samples tested, the mutant *Arf* allele was retained, as determined by RT-PCR (Supplemental Fig. S2); insertion of the *neo*-selectable marker used in the *Arf* targeting strategy results in a stop codon and no Arf protein expression (Fig. 3). *Arf*(+/−) mice showed loss of Ink4a expression in 3 of 6 MM cultures, whereas p15(Ink4b) was retained in 5 of 6 cultures (Fig. 3). Similarly, inactivation of Ink4a and p15(Ink4b) were not required for MM formation in a C57Bl/6 *Arf*(+/−) model [6].

MMs from *Ink4a*;*Arf* doubly heterozygous mice exhibited biallelic inactivation of *Ink4a* and *Arf*, whereas *Ink4b* was retained in 6 of 7 MM samples tested. Expression of Nf2 and p53 was retained in nearly all MM cultures from *Ink4a*(+/−), *Arf*(+/−), and *Ink4a*;*Arf*(+/−) mice (Fig. 3).

**Figure 4:**
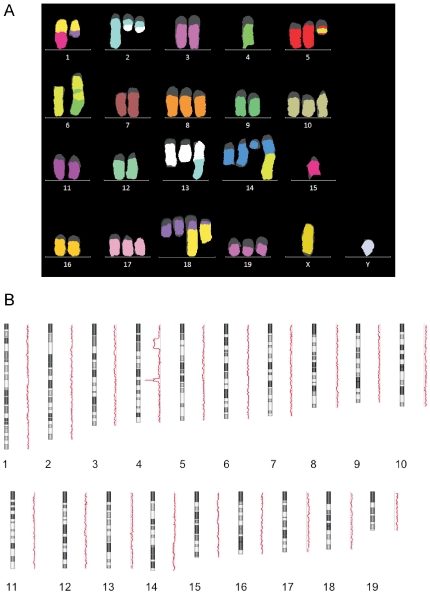
Chromosomal analyses of MM cells from an *Arf*-deficient mouse. *A*, mFISH revealed one or more copies of 1;15, 1;18, 2;13, 5;6, and 6;14 rearrangements; der(6) composed of (from centromere to telomere) segments of chromosomes 6, 4, 6, and 4; deletion of chromosome 14; and trisomies of chromosomes 8, 10, 17 and 19. *B*, aCGH analysis showed gains of chromosomes 8, 10, 13, 17, 18, and 19; and deletions of two regions of chromosome 4, one of which (*lower*) represents a homozygous deletion encompassing the *Cdkn2a/Arf* locus embedded within a hemizygous deletion.

Cytogenetic analyses were performed on two randomly-selected MM cultures from each mouse model. As in the human disease counterpart, MMs from *Ink4a*-, *Arf*-, and *Ink4a*;*Arf*-deficient mice typically showed numerous chromosome alterations. No consistent alteration was seen, although 3 of 6 tumors had extra copies of chromosome 19, including a MM from an *Ink4a*(+/−) mouse (#264), in which gain of chromosome 19 was the only abnormality observed. MM cultures from *Arf*- and *Ink4a*;*Arf*-deficient mice typically had numerous clonal structural rearrangements (Fig. 4A). Furthermore, aCGH analysis revealed a homozygous deletion encompassing the *Cdkn2a*/*Arf* locus (Fig. 4B).

## Discussion

Previous studies have shown that *Arf*(−/−) and *Ink4a*(−/−) mice are more prone to spontaneous tumors than wild-type animals, but each less so than *Ink4a*;*Arf*(−/−) mice [8]. The *in vivo* carcinogenesis studies reported here evaluated the contribution of heterozygous mutations of *Arf* and *Ink4a*, as well as a both tumor suppressor genes, to the induction of MM by asbestos, a well-established cause of this malignancy. *In vivo* genetic models were used to investigate the relative impact of *Arf* versus *Ink4a* deficiency in a common genetic background. By analogy, our findings suggest that *p14(ARF)*, like *p16(INK4A)*, is an important target of 9p21 deletions in human MM. Moreover, the data indicate that co-deletion of *Arf* and *Ink4a* can cooperate to accelerate tumorigenesis.

In previous studies of heterozygous *Arf* mice, spontaneous tumors exhibited loss of the residual wild-type *Arf* allele [2], consistent with a classical two-hit tumor suppressor gene. We found asbestos-induced MMs from heterozygous *Ink4a*, *Arf* and *Ink4a*;*Arf* mice required biallelic inactivation of the predisposing tumor suppressor genes, and MMs were detected faster in the doubly deficient model. The importance of these tumor suppressors is in accordance with an investigation using conditional knockout mice [9], in which adeno-Cre-mediated homozygous excision of *Ink4a*;*Arf* was sufficient to induce MM in the absence of asbestos exposure.

Notably, while tumor cells from *Ink4a*(+/−)-deficient mice acquired loss of Arf or p53 expression, loss of p16(Ink4a) was observed in only 3 of 6 MMs from *Arf*(+/−) mice. Similarly, in our earlier studies of *Arf*-deficient mice in a different (C57Bl/6) genetic background, all 11 MMs analyzed showed loss of Arf, although loss of p16(Ink4a) was identified in only two tumors. Collectively, these data imply that inactivation of Arf or p53 may be crucial for MM pathogenesis, whereas inactivation of p16(Ink4a) is not.

Since MMs analyzed here retained expression of p15Ink4b, loss of this gene is not critical for induction of MM by asbestos. In addition, our deletion mapping studies of human MMs revealed that deletions of *p15INK4B* occur less frequently than losses of *CDKN2A/ARF* and never occurred in the absence of a homozygous loss in the *CDKN2A/ARF* locus [5].

Also similar to our earlier study of *Arf*-deficient mice in a different (C57Bl/6) background, we rarely observed loss of Nf2 in MMs arising in *Ink4a*-, *Arf*- or *Ink4a*;*Arf*-deficient mice. Inactivation of the *NF2* tumor suppressor gene is postulated to facilitate cell cycle progression and tissue invasion/metastasis [10], and *Nf2*-deficient mice are predisposed to asbestos-induced MM and contribute to its invasiveness and spreading [5], [11]. However, NF2/merlin loss does not appear to be required for development of MM in mice having this genotype and/or background.

Lastly, we found retention of functional p53 expression in MM cells that exhibit loss of Arf expression. Only one of the 20 asbestos-induced MMs showed loss of p53 expression (Fig. 3B), and that single sample retained expression of Arf, consistent with our previous work showing a reciprocal pattern of inactivation of Tp53 in asbestos-induced MMs from *Nf2*(+/−) mice that had retained expression of Arf [5], [6]. Interestingly, in MM cells with loss of Arf, the p53 pathway appeared to remain functional based on response to DNA damage (Fig. 3C). These results from genetic model systems suggest that Arf loss can contribute to MM pathogenesis via p53-independent pathway(s), as previously noted in human MM cells [12], and that an intact p53 pathway remains a potential target for the treatment of this highly aggressive, chemo-resistant malignancy.

In summary, this is the first report directly assessing the relative importance of *Ink4a* and *Arf* in the susceptibility to asbestos-induced MM. Collectively, these *in vivo* data indicate that both *Cdkn2A/Arf* gene products suppress asbestos carcinogenicity. Furthermore, while Arf inactivation appears to be critical for MM pathogenesis and genomic instability (Fig. 4), the inactivation of both *p16(Ink4a)* and *p19(Arf)* cooperate to accelerate asbestos-induced tumorigenesis. Thus, future therapeutic approaches for MM should consider targeting pathways cooperatively regulated by both tumor suppressor genes.

## Materials and Methods

### Animals and treatments

*Ink4a* (01XE4, FVB.129-*Cdkn2a^tm2.1Rdp^*) [3] and *Ink4a*;*Arf* (01XB2, FVB/N.129-*Cdkn2a^tm1Rdp^*) [4] mice were from the Mouse Models of Human Cancers Consortium. Mice lacking *Arf* (a gift of N. Sharpless) were generated with Cre-mediated excision of the neomycin selection cassette, similar to *Ink4a* mice [3]. All mice were in a comparable genetic background [8]. Mice were backcrossed at least two additional generations with FVB/N mice for uniformity and genotyped as described (MMHCC and [8]). Procedures were compliant with the NIH *Guide for the Care and Use of Laboratory Animals*.

6–8 week-old mice were injected intraperitoneally every 3 weeks with 400 µg crocidolite (UICC, SPI Supplies) (total, 3.2 mg/mouse), or with equivalent control TiO_2_ particles (Aldrich) [5], [6]. Mice were scored as having MM based on histological evidence and/or if tumor cells exhibited a combination of three or more MM markers, including mesothelin, as assessed by reverse transcriptase-PCR (RT-PCR) and/or immunohistochemistry (Supplemental tables in File S1, Supplemental Fig. S1). This study was carried out in strict accordance with the recommendations in the Guide for the Care and Use of Laboratory Animals of the National Institutes of Health. The protocol was approved by the Committee on the Ethics of Animal Experiments of the Fox Chase Cancer Center (protocol number: 00-26). All surgery was performed under sodium pentobarbital anesthesia, and all efforts were made to minimize suffering.

### Immunohistochemistry

Slides of formalin-fixed, paraffin-embedded samples were incubated with antibodies against pan-cytokeratin and cytokeratin 8 (Sigma) and mesothelin (Santa Cruz Biotechnology), which were detected with biotinylated secondary antibodies. Sections were stained with DAB and counterstained with hematoxylin.

#### Primary cell cultures

Primary MM cells were isolated from ascitic fluid and/or peritoneal lavage, as described [5]. All primary cell cultures used for the molecular analyses were from passages ≤6. PCR analysis was conducted on all cultures that expressed mesothelial markers, and immunoblot analysis was performed on a random set of cultures to validate the PCR results. To test for p53, sub-confluent mouse MM cells (>passage 6) were treated with Etoposide (100 µM) or UV irradiation (80 J/m^2^) and harvested 24 hours post-treatment for immunoblotting.

### PCR

RT-PCR was used to evaluate tumor cells for expression of E-cadherin, N-cadherin, cytokeratin 18 and cytokeratin 19 [5]. Control *Gapdh* was used to assess template integrity [6]. RT-PCR for mesothelin used primers 5′-ATCAAGACATTCCTGGGTGGG-3′ and 5′-CGGTTAAAGCTGGGAGCAGAG-3′. Oligonucleotides for genomic and RT-PCR of *Ink4a*, *Ink4b*, *Nf2* and *p53* were as described [5], [6]. Primers for *p19(Arf)* exon 1β were based on National Center of Biotechnology Information (NCBI) sequences.

### Immunoblotting

Immunoblots were prepared with 15–30 µg of protein/sample, as described [5], [6]. Antibodies included anti-Arf (Abcam), anti-p53 (NCL-p53-505, Novocastra), and anti-Ink4a (M-156), -Nf2 (H-260), and -β-actin (I-19) (Santa Cruz).

### Karyotypic and M-FISH Analysis

Preparation of metaphases and G-banding were performed as reported [13]. Guidelines for karyotypic designations of mouse metaphase chromosomes are found at http://www.pathology.washington.edu/research/cytopages/idiograms/mouse/. Metaphase preparations were hybridized using a 21Xmouse mFISH kit (MetaSystems). Image capturing/processing utilized a Zeiss AxioImager Z2 fluorescence microscope, with single band pass filters (Chroma Technology) appropriate for each fluorochrome and an Isis/mFISH image analysis system (MetaSystems).

### Array-CGH

Genomic DNA was isolated from primary MM cell cultures at passages ≤6. Agilent 244K Genomic DNA Arrays and scanner were used for DNA copy number analysis. Data were extracted using Feature Extraction Software, and output was imported into CGH Analytics for DNA Copy Number Analysis (Agilent).

## Supporting Information

Figure S1 Immunohistochemical staining of a MM tumor with anti-mesothelin (MSN) or anti-MSN plus blocking peptide to show specificity of staining.
(TIF)

Figure S2 Retention of mutant *Arf* allele in MMs from *Arf* (+/−) mice. *A*, Abnormally large RT-PCR product amplified with *Arf*-specific primers for exon 1β. Samples are from MM cells of five *Arf* (+/−) mice (lanes 1–5); lane 6 is from wild-type mouse embryonic fibroblasts. *B*, Sequencing of PCR products revealed an 84-bp insertion (grey italicized letters) in the mutated *Arf* allele (Mu.) replacing an AG (underlined) in the wild-type allele (Wt.). The insertion generates a predicted stop codon (marked in black), which would result in unsuccessful translation of the p19(Arf) protein.
(TIF)

File S1 MM Markers for primary cell cultures derived from asbestos-treated mice.
(DOC)
