# Supplementary figures and images for: Losses of Both Products of the Cdkn2a/Arf Locus Contribute to Asbestos-Induced Mesothelioma Development and Cooperate to Accelerate Tumorigenesis
Source: PLoS One. 2011 Apr 19;6(4):e18828. doi: 10.1371/journal.pone.0018828 (PMC3079727; doi:10.1371/journal.pone.0018828)

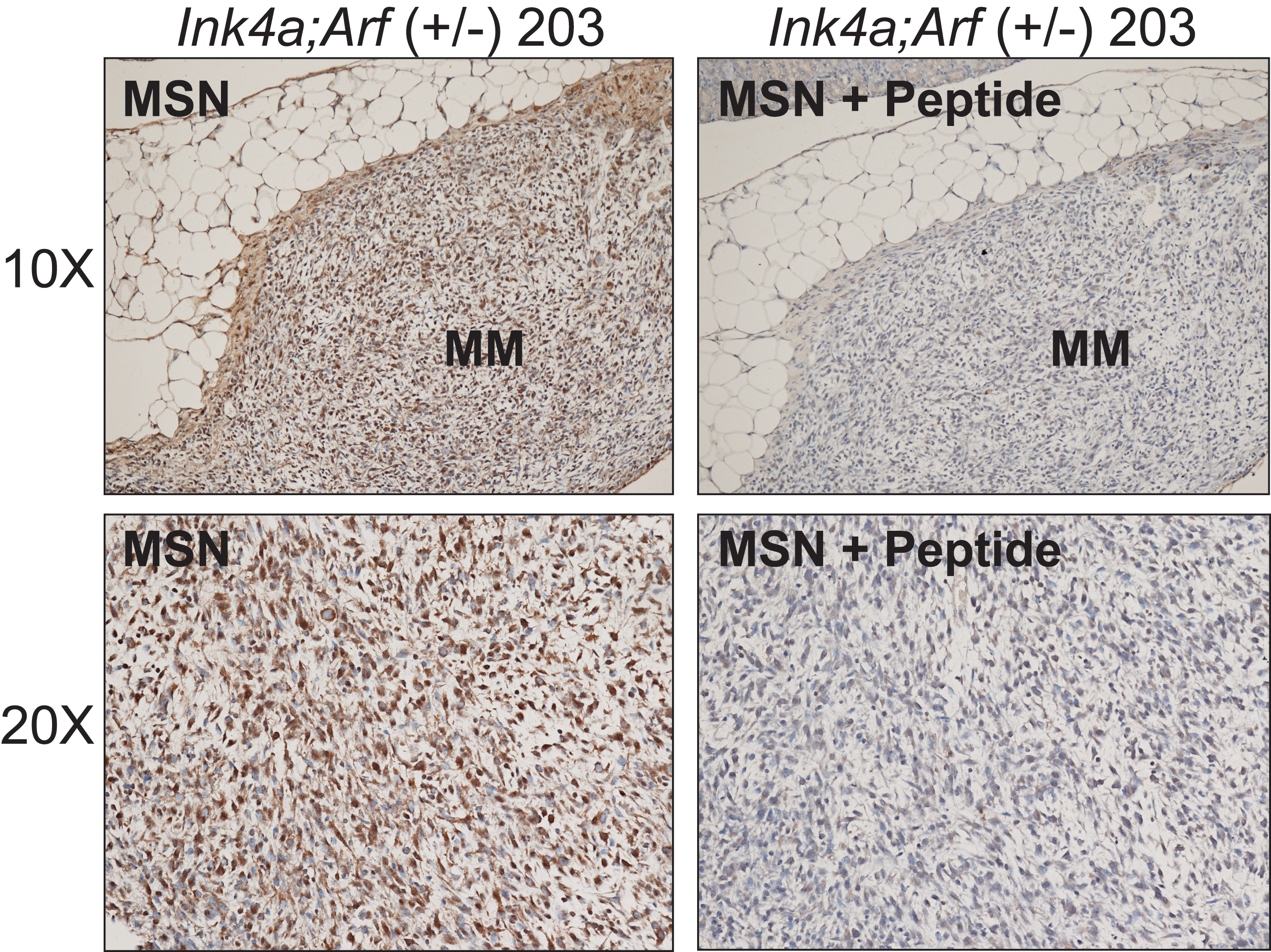

Supplement: Figure S1 — Immunohistochemical staining of a MM tumor with anti-mesothelin (MSN) or anti-MSN plus blocking peptide to show specificity of staining. (TIF) [file pone.0018828.s001.tif]

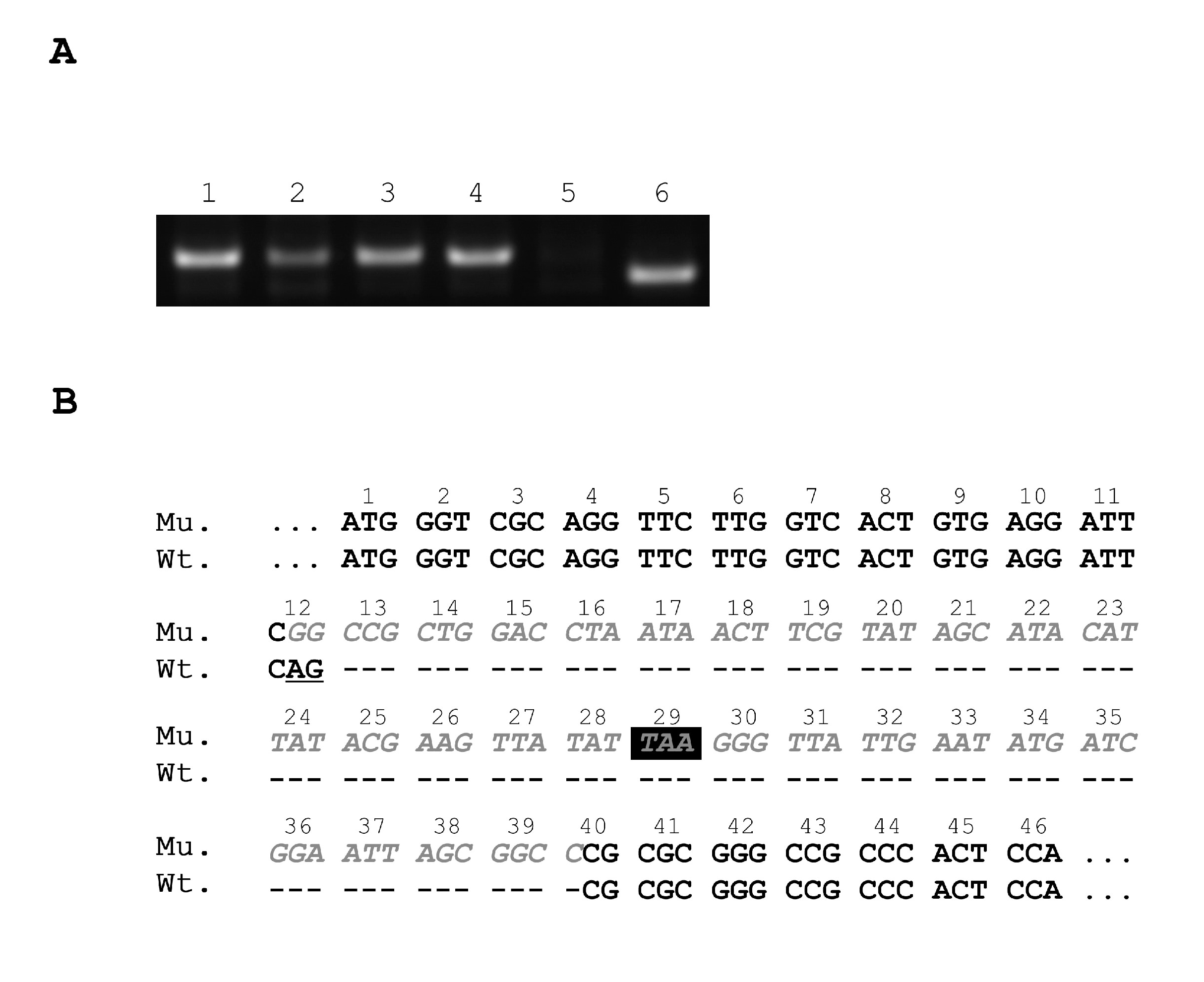

Supplement: Figure S2 — Retention of mutant Arf allele in MMs from Arf (+/−) mice. A, Abnormally large RT-PCR product amplified with Arf-specific primers for exon 1β. Samples are from MM cells of five Arf (+/−) mice (lanes 1–5); lane 6 is from wild-type mouse embryonic fibroblasts. B, Sequencing of PCR products revealed an 84-bp insertion (grey italicized letters) in the mutated Arf allele (Mu.) replacing an AG (underlined) in the wild-type allele (Wt.). The insertion generates a predicted stop codon (marked in black), which would result in unsuccessful translation of the p19(Arf) protein. (TIF) [file pone.0018828.s002.tif]
