# Supplementary material for: Losses of Both Products of the Cdkn2a/Arf Locus Contribute to Asbestos-Induced Mesothelioma Development and Cooperate to Accelerate Tumorigenesis
Source: PLoS One. 2011 Apr 19;6(4):e18828. doi: 10.1371/journal.pone.0018828 (PMC3079727; doi:10.1371/journal.pone.0018828)
Supplement: File S1 — MM Markers for primary cell cultures derived from asbestos-treated mice. (DOC) [file pone.0018828.s003.doc]

**Supplemental Table S1.** MM Markers for Primary Cell Cultures Derived from Asbestos-Treated Mice

| **Genotype** | **Mouse** | **MM pathology** | **Mesothelin** | **E-cadherin** | **N-cadherin** | **Cytokeratin18** | **Cytokeratin19** |
| --- | --- | --- | --- | --- | --- | --- | --- |
| wt | 299 | + | + | + | + | + | + |
| wt | 323* | + | + | - | + | + | - |
| *Ink4a* (+/-) | 196 | + | + | + weak | + weak | + | + |
| *Ink4a* (+/-) | 200* | + | + | - | - | - | - |
| *Ink4a* (+/-) | 247 | + | + | - | + | + | + weak |
| *Ink4a* (+/-) | 248 | - | + | + weak | + | + | + |
| *Ink4a* (+/-) | 264 | - | + | + | + | + | + |
| *Arf* (+/-) | 239 | - | + | + weak | - | + | + |
| *Arf* (+/-) | 275 | + | + | - | + | + | + weak |
| *Arf* (+/-) | 304 | + | + | - | + | + | +weak |
| *Arf* (+/-) | 307 | - | + | - | + | +weak | - |
| *Arf* (+/-) | 314* | + | + | + | + | - | + |
| *Arf* (+/-) | 426 | - | + | - | + | + | + |
| *Ink4a*;*Arf* (+/-) | 49 | + | + | + | + | + | + |
| *Ink4a*;*Arf* (+/-) | 59 | + | + | - | + | - | + |
| *Ink4a*;*Arf* (+/-) | 77 | - | + | - | + | + | - |
| *Ink4a*;*Arf* (+/-) | 81 | - | + | - | + | + | + |
| *Ink4a*;*Arf* (+/-) | 120 | - | + | - | + | + | + |
| *Ink4a*;*Arf* (+/-) | 202 | + | + | + | + | + | + |
| *Ink4a*;*Arf* (+/-) | 203* | + | + | - | - | - | - |

* MM positive for cytokeratin based on immunohistochemical staining.

**Supplemental Table S2.** Summary of Asbestos-Treated Mice

|  | *Ink4a*;*Arf* (+/-) | *Ink4a* (+/-) | *Arf* (+/-) | Wild-type |
| --- | --- | --- | --- | --- |
| # mice i.p. asbestos | 36 | 38 | 32 | 20 |
| # mice collected | 33 | 29 | 26 | 14 |
| % mice w MM | 29/33 (88%) | 19/29 (66%) | 17/26 (65%) | 7/14 (50%) |
| Median MM latency post- asbestos (wks) | 29.6 | 34.6 | 38.0 | 49.4 |
| % mice w ascites | 30/33 (88%) | 21/29 (72%) | 20/26 (77%) | 10/14 (71%) |
| Other tumors | 1 with invasive MM in esophagus; 2 with lung adenoma | 1 lung adenocarcinoma | 3 with lung adenocarcinoma; 2 with lung adenoma | None |

**Supplemental Table S3.** Summary of Control Ti0_2_-Treated *Ink4a*;*Arf* (+/-) Mice Aged > 1 year

|  | Injected w/ i.p. TiO_2_ | No Treatment |
| --- | --- | --- |
| # mice | 13 | 9 |
| # without tumors | 10 | 8 |
| Spontaneous tumors | 1 lymphoma  1 lung adenocarcinoma  1 skin squamous papilloma | 1 poorly differentiated tumor affecting ovary, uterus & liver |
